# Measuring health inequalities: a systematic review of widely used indicators and topics

**DOI:** 10.1186/s12939-021-01397-3

**Published:** 2021-03-10

**Authors:** Sergi Albert-Ballestar, Anna García-Altés

**Affiliations:** 1 Catalan Health System Observatory, Agència de Qualitat i Avaluació Sanitàries de Catalunya (AQuAS), 81-95 (2a planta), 08005 Barcelona, Spain; 2 CIBER de Epidemiología y Salud Pública (CIBERESP), Barcelona, Spain; 3 Institut d’Investigació Biomèdica (IIB Sant Pau), Barcelona, Spain

**Keywords:** Health inequalities, Health indicators, Review, Health policy

## Abstract

**Background:**

According to many conceptual frameworks, the first step in the monitoring cycle of health inequalities is the selection of relevant topics and indicators. However, some difficulties may arise during this selection process due to a high variety of contextual factors that may influence this step. In order to help accomplish this task successfully, a comprehensive review of the most common topics and indicators for measuring and monitoring health inequalities in countries/regions with similar socioeconomic and political status as Catalonia was performed.

**Methods:**

We describe the processes and criteria used for selecting health indicators from reports, studies, and databases focusing on health inequalities. We also describe how they were grouped into well-known health topics. The topics were filtered and ranked by the number of indicators they accounted for.

**Results:**

We found 691 indicators used in the study of health inequalities. The indicators were grouped into 120 topics, 34 of which were selected for having five indicators or more. Most commonly found topics in the list include “Life expectancy”, “Infant mortality”, “Obesity and overweight (BMI)”, “Mortality rate”, “Regular smokers/tobacco consumption”, “Self-perceived health”, “Unemployment”, “Mental well-being”, “Cardiovascular disease/hypertension”, “Socioeconomic status (SES)/material deprivation”.

**Conclusions:**

A wide variety of indicators and topics for the study of health inequalities exist across different countries and organisations, although there are some clear commonalities. Reviewing the use of health indicators is a key step to know the current state of the study of health inequalities and may show how to lead the way in understanding how to overcome them.

## Introduction

Strong efforts to tackle health inequalities can be seen at international and national level since the 1980s. In early 2008, the World Health Organization’s (WHO) Global Commission on Social Determinants of Health called for action on the social determinants of health, the conditions in which persons are born, grow, work, live, and age, to “close the gap in a generation” [1]. In late 2008, the Spanish Public Health General Direction (*Dirección General de Salud Pública)* and the Foreign Health of Health Ministry and Social Policy (*Sanidad Exterior del Ministerio de Sanidad y Política Social*) requested the constitution of the Commission for the Reduction of Social and Health Inequalities (*Comisión para Reducir las Desigualdades Sociales en Salud en España* (CRDSS-E) [2]. The mission of CRDSS-E was to elaborate on a proposal of intervention measures to reduce health inequalities. The CRDSS-E published two documents: one analysing health inequalities in the Spanish context [3], and another describing some policy proposals to tackle them [4]. In 2011, a total of 125 countries, Spain being one of them, developed and signed the Rio Political Declaration on Social Determinants of Health [5]. The declaration recommended interventions from governments and international organisations [6].

At a regional level in Catalonia, tackling health inequalities is one of the main goals of both the Catalan Health Plan 2016–2020 (led by the Health Department of the Catalan Government) [7] and the Interdepartmental and Intersectorial Public Health Plan 2017–2020 (PINSAP) [8, 9]. During the past years, various reports and peer-reviewed papers about the health effects of the economic crisis on the population of Catalonia were published by the Catalan Health System Observatory [10–17].

Overall, much effort has been devoted to monitoring and tackling health inequalities at regional, national, and international levels. Even so, OECD countries continue to present large disparities in health, including, for example, significant differences in life expectancy between people with the highest and lowest levels of education [18]. The selection of topics represents the first step in monitoring health inequalities according to many conceptual frameworks and is highly relevant, as these topics will potentially limit the detection of health inequalities within the population, hence playing a key role in providing evidence for posterior decision-making [19, 20]. Yet some difficulties may arise during the selection of relevant topics, as well as their health indicators. A wide diversity of indicators for monitoring health inequalities have been used across different countries and organisations; this is due to the high variety of contextual factors that may have an influence on it, such as the study goals or the information resources available.

In order to help accomplish this task successfully, the objective of this study is to perform a systematic review of the most common topics and indicators used for measuring and monitoring health inequalities in the reports, projects, and databases of international, national, and regional governmental organizations.

The main purpose of this study is to provide a broad overview of health inequalities topics considered relevant by different public health organizations. Nevertheless, the focus of this review is on countries/regions with similar socioeconomic and political status to Catalonia. It may also be useful for other organizations who decide to study or monitor health inequalities to accomplish its very first step: topic selection. In addition, gaining some insights about which health issues are being prioritized, as well as which indicators were used, are considered secondary goals.

## Material and methods

First, a bibliographic search was performed using PubMed, Google Scholar, and Google search engine with the terms “*health inequalities*”, “*health observatories*” and “*health inequalities indicators*”. Occasionally, names of concrete regions, countries, or organisations were added to these terms (i.e., “*Andalucía health observatory*” or “*Canada health inequalities*”). The search was performed from March to June of 2019. Once finished, a set of inclusion criteria was applied; studies included in the review had to:

1. **Include health inequalities indicators**: All the reports that contained no health indicators were automatically discarded (i.e., policy frameworks [21]).
2. **Have been carried out by a governmental organisation or a related entity, whether at an international, national, or regional level**: the reports not published by governmental (or government-related) organisations were discarded.
3. **Have a socioeconomic and political status similar (or highly related) to Catalonia**: some reports were discarded due to significant differences in the socioeconomic profile of the countries they were studying in comparison to Catalonia or Spain.

Once the reports were selected, the authors performed a quality control check of the indicators shown in the reports and databases. The indicators had to match the basic anatomy of an indicator as a minimum requirement to be considered an indicator. This basic anatomy consists of containing data, i.e. the numerical data input; and containing good metadata, like a title and an explanation of how an indicator is defined and calculated [22]. In addition, the different reports found were classified according to the geo-political region they were studying: 1. international, 2. national, and 3. regional (Table 1).

After this selection process, the indicators were grouped into topics by semantic matching of their definition as well as by the area of knowledge there are intended to measure. Most of the topics were supported by references of relevant organisations like the WHO or the United Nations (UN) (see Table 2). Each topic was uniquely named in accordance with the area of knowledge that instruments were intended to measure. Indicators from different sources were often merged due to high similarities between them (most commonly, the only differences were stratifiers such as age, gender or region). Every topic had to be formed by at least five indicators in order to be considered relevant enough; the topics with less than five indicators were discarded.

The search was performed by the two researchers, and the results shared in order to agree on any discrepancies. All the data was organised in spreadsheets to identify common indicators, and then sorted by the amount of indicators they included. The names of the indicators in the spreadsheets were those given in the original reports or their metadata information.

## Results

In total, 21 reports, projects, and databases were identified and classified into three categories: 1. international [8], 2. national [10], and 3. regional [3]. In the first category, international, all the projects selected were carried out or funded by the European Commission [23–27], the WHO [28, 29] or the World Bank [30]. In the following category, national, studies were conducted by health agencies or governments of countries such as Andorra [31], Australia [32], Canada [33, 34], England [35, 36], Scotland [37], Slovenia [38], Spain [39] or Portugal [40]. In the last category, regional, some reports published by Spanish regions were included (Andalucía [41], Barcelona [42], Valencia [43]). A total of 691 health indicators were identified (Table 1).

**Table 1:** Reports and datasets identified in the search

Type	Organisation/ Institution	Report or database title	Number of indicators	URL for website or PDF	Reference
International	WHO Regional Office for Europe	Data Management Tool	6	http://dmt.euro.who.int/classifications/tree/B#B03	[28]
International	European Commission	Health inequalities in the EU	12	https://ec.europa.eu/health/sites/health/files/social_determinants/docs/healthinequalitiesineu_2013_en.pdf	[25]
International	Social Protection Committee Indicators Sub-group (European Commission)	Portfolio of EU Social Indicators for the Monitoring of Progress Towards the EU Objectives for Social Protection and Social Inclusion	12	https://ec.europa.eu/social/BlobServlet?docId=14239&langId=en	[26]
International	Various organisations (European Commission funded project)	I2SARE	37	https://www.sergas.es/Saude-publica/-I2SARE-Galicia	[27]
International	European Commission	European Core Health Indicators (ECHI)*	64	https://ec.europa.eu/health/indicators/echi/list_en#id3	[24]
International	European Commission	Eurostat (SDG)	13	https://ec.europa.eu/eurostat/web/sdi/good-health-and-well-being	[23]
International	The World Bank	World Bank Open Data (Indicators section)	52	https://data.worldbank.org/indicator/	[30]
International	WHO	100 Core Health Indicators (plus health-related SDGs) 2018	100	https://www.who.int/healthinfo/indicators/100CoreHealthIndicators_2018_infographic.pdf?ua=1	[29]
National	Institute of Health Equity	Marmot Indicators Release 2017	10	http://www.instituteofhealthequity.org/about-our-work/marmot-indicators-release-2017	[35]
National	Canadian Institute for Health Information	Health Inequalities Map	105	https://infobase.phac-aspc.gc.ca/health-inequalities/docs/health-inequalities-map-en.pdf	[34]
National	Public Health Agency of Canada (PHAC)	Key Health Inequalities in Canada: A National Portrait – Executive Summary	22	https://www.canada.ca/en/public-health/services/publications/science-research-data/key-health-inequalities-canada-national-portrait-executive-summary.html	[33]
National	Government of Scotland	Long-term monitoring of health inequalities: December 2018 report	14	https://www2.gov.scot/Publications/2018/12/8085	[37]
National	National Health Service (England)	England Analysis: NHS Outcome Framework Health Inequalities Indicators 2016/17	30	https://www.england.nhs.uk/wp-content/uploads/2017/07/nhs-outcome-framework-health-inequalities-indicators-2016-17.pdf	[36]
National	*Institut d’Estadística d’Andorra*	*Observatori Social d’Andorra*	13	https://observatorisocial.ad/	[31]
National	Australian Institute of Health and Welfare	Health inequalities in Australia: morbidity, health behaviours, risk factors, and health service use	20	https://www.aihw.gov.au/getmedia/0cbc6c45-b97a-44f7-ad1f-2517a1f0378c/hiamhbrfhsu.pdf	[32]
National	WHO Regional Office for Europe	Health Inequalities in Slovenia	32	http://www.euro.who.int/__data/assets/pdf_file/0008/131759/Health_inequalities_in_Slovenia.pdf	[38]
National	*Instituto Nacional de Estadística (Portugal)*	*Indicadores Sociais - 2011*	16	https://censos.ine.pt/xportal/xmain?xpid=INE&xpgid=ine_publicacoes&PUBLICACOESpub_boui=149279938&PUBLICACOEStema=55538&PUBLICACOESmodo=2	[40]
National	Ministry of Health and Social Policy of Spain	Moving Dorward Equity in Health: Monitoring Social Determinants of Health and the Reduction of Health Inequalities	30	https://www.mscbs.gob.es/profesionales/saludPublica/prevPromocion/promocion/desigualdadSalud/PresidenciaUE_2010/conferenciaExpertos/docs/haciaLaEquidadEnSalud_en.pdf	[39]
Regional	*Observatorio Valenciano de la Salud*	*Desigualdades en Salud en la Comunidad Valenciana*	26	https://www.sp.san.gva.es/DgspPortal/docs/20180301_Desigualdades_Salud_OVS2018.pdf	[43]
Regional	*Ajuntament de Barcelona*	*Desigualtats en salut, respostes a nivell local: Polítiques per reduir les desigualtats en salut a la ciutat de Barcelona*	22	http://www.consorci.org/media/upload/arxius/coneixement/salut-publica/2017/D_%20Malmusi_Desigualtats_28-09-2017.pdf	[42]
Regional	*Escuela Andaluza de Salud Pública*	*Guía de indicadores para medir las desigualdades de género en salud y sus determinantes*	55	https://www.easp.es/project/guia-de-indicadores-para-medir-las-desigualdades-de-genero-en-salud-y-sus-determinantes/	[41]

**Fig. 1:**
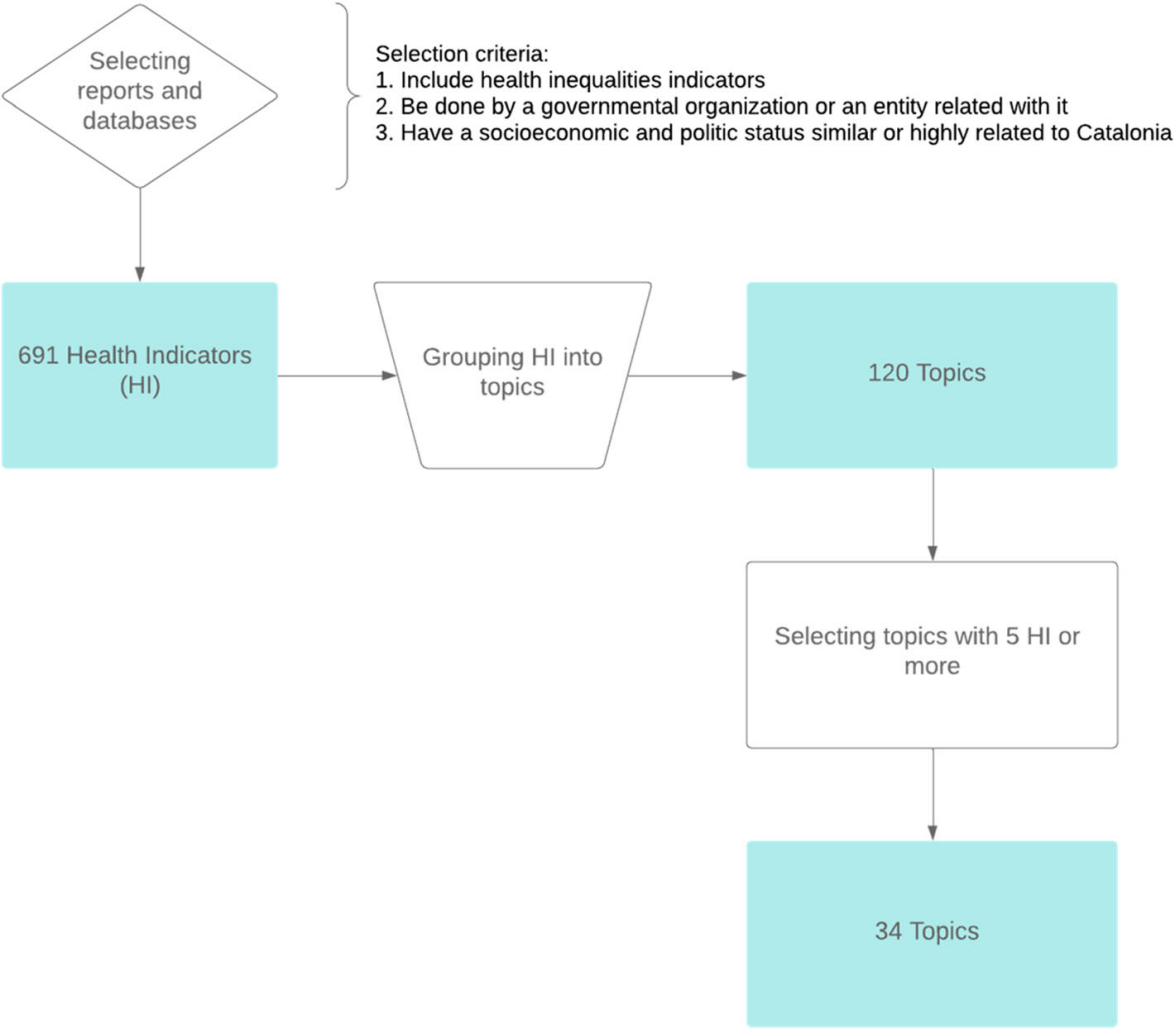
Flowchart of the processes undertaken to review the most common topics in the study of health inequalities

Following an iterative process of evaluation, we identified a core set of 120 candidate topics, of which 34 were finally selected (Fig. 1). Table 2 describes a complete list of 34 topics with the corresponding definition of each topic. The ten most commonly used were: “Life expectancy”, “Infant mortality”, “Obesity and overweight (BMI)”, “Mortality rate”, “Regular smokers/tobacco consumption”, “Self-perceived health”, “Unemployment”, “Mental well-being”, “Cardiovascular disease/hypertension”, “Socioeconomic status (SES)/material deprivation”. However, some topics that ranked below these were closely related with some of the most common topics; for example, “Perinatal, neonatal and stillbirths mortality” might be considered as a subtype of “Infant mortality”; and “Perceived mental health” is similar to both “Mental well-being” and “Self-perceived health”. Furthermore, some indicators may represent antithetically the same area of knowledge; that is the case for the indicators in the topic “Long-term limitations/chronic illnesses” and the indicator “Healthy Life Years (HLY)” [within the topic “Life expectancy”], where the former (long-term limitations or chronic illnesses) is used to determine the latter (the end of a healthy life condition). Nevertheless, the metadata of the health indicators within each topic was highly homogeneous: they all had similar definitions and methodology.

**Table 2:** List of topics ranked by the number of health indicators grouped within

Topic	Description of measured topics	Reference
Life expectancy	Time that a concrete population is expected to live.	[23–30, 33–39, 41–43]
Infant mortality	Death of young children under the age of 1 in a population.	[24–30, 33, 34, 36, 38–42]
Obesity and overweight (BMI)	Measurement of people having more body fat than is optimally healthy, to an extent that it may have a negative effect on health.	[22, 24, 25, 27, 29–33, 36, 38, 42, 43]
Mortality rate	Measurement of the number of deaths in a particular population, scaled to the size of that population, per unit of time.	[25–27, 29, 30, 34, 37, 38, 40–43]
Regular smokers/ tobacco consumption	Measurement of the number of people consuming tobacco (smoking) in a population.	[23–25, 27, 29, 31–34, 38, 42, 43]
Self-perceived health	Measurement of the expression of subjective assessment by the respondent of his/her health.	[23–26, 31, 32, 34, 36–38, 42, 43]
Unemployment	Measurement of people above a specified age that are not in paid employment or self-employment and are currently available for work during the reference period.	[24, 25, 27, 28, 30, 34, 36, 38, 39, 41, 42],
Mental well-being	Level of psychological well-being or an absence of mental illness.	[24, 29, 33, 34, 36–39, 41–43]
Cardiovascular disease/ hypertension	Measurement of the number of people affected by cardiovascular disease (CVD) or hypertension in a population.	[24, 27, 29, 32, 34, 36–38, 41, 43]
Socioeconomic status (SES)/ material deprivation	Measurement of the social standing or class of individuals in a population, as well as the state of economic strain and durables.	[24, 25, 28, 33–35, 38, 39, 42],
Diabetes/ insulin resistance	Measurement of people affected by diabetes (a chronic, metabolic disease characterised by elevated levels of blood glucose) or insulin resistance in a population.	[24, 25, 29, 30, 32–34, 38, 41],
Physical activity	Measurement of physical activity (any bodily movement produced by skeletal muscles that requires energy expenditure) in a population.	[24, 29, 31, 32, 34, 38, 39, 42],
Cancer	Measurement of people affected by cancer (group of diseases that can start in almost any organ or tissue of the body when abnormal cells grow uncontrollably, go beyond their usual boundaries to invade adjoining parts of the body and/or spread to other organs) in a population.	[24, 25, 27, 33, 34, 36, 37, 43]
HIV	Measurement of people affected by HIV (an infection that attacks the body’s immune system, specifically the white blood cells, called CD4 cells) in a population.	[23, 24, 27, 29, 30, 34, 40, 41],
Long-term limitations/ chronic illnesses	Measurement of people affected by diseases or conditions that are persistent or long-lasting.	[23–26, 31, 34, 36, 37]
Tuberculosis	Measurement of people with health issues caused by the infection of the bacteria *Mycobacterium tuberculosis* in a population.	[23, 29, 30, 33, 34, 40, 42, 43]
Hazardous alcohol consumption	Measurement of hazardous alcohol consumption in a population.	[24, 29, 32–34, 37, 38]
Low birthweight	Measurement of birth weights of infants of 2499 g or less in a population.	[27, 29, 34, 37, 38, 41, 42],
Perinatal, neonatal and stillbirths mortality	Measurement of foetus or neonate deaths in a population.	[24, 27, 29, 30, 36, 38, 43]
General practitioner (GP) utilisation	Measurement of the utilisation of general practitioners (medical doctors) in a population.	[24, 26, 31, 32, 34, 36, 39]
Suicide/self-harm	Measurement of the number of intentionally self-caused deaths and/or other intentional self-harm injuries in a population.	[25, 29, 33, 34, 36, 38, 43]
Healthcare resources	Measurement of resources such as materials, personnel or facilities that can be used to provide healthcare.	[24, 27, 30, 38–40, 43]
Alcohol consumption	Measurement of people consuming alcohol (a toxic and psychoactive substance with dependence-producing properties) regularly in a population.	[24, 25, 31, 32, 37, 38]
Road traffic accidents (injuries and deaths)	Measurement of deaths and injuries due to road traffic accidents (crashes) in a population.	[23, 24, 27, 29, 30, 38],
Food consumption (vegetables, fruit, salt)	Measurement of people ingesting solid foods in a population.	[24, 29, 32, 34, 38, 39]
Primary studies/ illiteracy	Measurement of people with primary studies (first stage of formal education) or illiterate (not able to read) in a population.	[24, 28, 32, 35, 39, 42]
Child well-being	Indicators to measure the general health, proper growth, and well-being of children.	[31, 33, 35, 39–41],
Respiratory disease	Measurement of people affected by a disease affecting the organs and tissues that make gas exchange in a population.	[24, 29, 32, 36, 38, 41],
Work-related health risks	Measurements of risks and/or diseases as a result of an exposure to risk factors from work activity in a population.	[23, 24, 29, 36, 39, 43]
Dental care/ oral health	Measurement of people with oral diseases or provided with dental health services in a population.	[26, 32–34, 36, 38],
Policy and legislation	Indicators measuring policy-making initiatives.	[24, 28, 29, 36, 39, 40]
Perceived mental health	Measurement of the expression of subjective assessment by the respondent of his/her mental health and/or psychological well-being.	[24, 30, 33, 34, 36]
Unintentional injuries	Measurement of people affected by any injury that is not caused on purpose or with intention to harm in a population.	[24, 33, 36, 38, 41],
Pregnancy care/ breastfeeding	Indicators measuring the care provided during pregnancy or other pregnancy-related issues, such as breastfeeding, in a population.	[24, 32, 34, 39, 41],
Hip fractures and surgical procedures	Measurement of the amount of surgical procedures and/or relevant fractures that will likely require surgical procedures (i.e. hip fractures) in a population.	[24, 30, 36, 41, 43]

In general, the indicators within each topic were very similar. In fact, often the differences among them were related to the different stratifiers (such as sex, age or region) used for their calculation. For example, in the first topic “Life expectancy”, indicators have different variations: “Life expectancy at birth” [23–25, 27–29, 32, 33, 37, 40–42], “Life expectancy at birth by sex” [22, 24–27, 33, 34], “Life expectancy at a certain age” [25, 33, 35, 40], “Life expectancy by educational attainment level” [25], and “Life expectancy at birth by socioeconomic status” [25]. Complex measures of inequality, such as “Slope index of inequality (SII) for male and female life expectancy” [34] may be considered another (more advanced) variation.

Furthermore, in the case of a health outcomes or diagnostics (such as mortality or cancer) the concrete disease or cause may also play an important role in the heterogeneity found among the health indicators. Indicators are focused on different aspects, such as prevalence and incidence, mortality, preventive measures, or treatments. For example, for the topic “HIV”: “HIV incidence” [23, 26, 28, 33, 40], “Prevalence of HIV, male/female, by ages” [29, 39] and “AIDS-related mortality rate” [28, 39] are the most common, yet “Antiretroviral therapy (ART) coverage” [28] and “HIV test results for TB patients (positive results)” [28] can also be relevant.

The results of the study show that the most common topics are related to:

- Mortality/life expectancy: “Life expectancy” [44, 45], “Infant mortality” [46] or “Mortality rate” [47] are widely used to study health inequalities.
- Incidence/mortality rates of specific diseases: “Cardiovascular disease/hypertension”, “Cancer (incidence or mortality)” [48], “Diabetes/insulin resistance” [49], “HIV” [50], “Tuberculosis (TB)” [51] or “Respiratory diseases”.
- Social determinants of health [52]:
  - ◦ “Living and working conditions” where this could be studied at an individual level, was highly ranked: “Unemployment” [53] and “Primary studies/illiteracy”. Otherwise, these indicators were at the bottom of the list.
  - ◦ This was similar for “Individual lifestyle factors and social and community networks” topics, which can also be studied at an individual level: “Obesity and overweight (BMI)” [54, 55], “Regular smokers/tobacco consumption” [56], “Alcohol consumption” [57], “Hazardous alcohol consumption”, “Physical activity” [58], and “Food consumption (vegetables, fruit, salt)”.
  - ◦ Socioeconomic level: “Socioeconomic status (SES)/material deprivation” [59, 60].
  - ◦ Healthcare system: “Healthcare resources” and “Policy and Legislation”.

## Discussion

### Main results of the study

The results of the study showed that the most common topics were related to mortality/life expectancy, incidence/mortality rates of specific diseases (i.e., TB or HIV), and social determinants of health, such as living and working conditions, and individual lifestyle factors and social and community networks, according to Dahlgren and Whitehead’s model of the social determinants of health [52]. The indicators that can be studied at an individual level tended to be highly ranked, in comparison to those that are studied at different levels (such as hospital or region), which tended to be at the bottom. Many methodological differences between indicators were due to stratifiers in their calculation.

### Study selection criteria

*To include health inequalities indicators* was a fundamental requirement for any report in order to be included in the review. Hence, although some reports provided in-depth insights about tackling health inequalities (i.e., [21, 61]) they were not selected due to lack of health indicators monitoring.

In addition, *to be carried out by a governmental or a government-related organisation* was also an important requirement, as many academic and/or private institutions carry out studies and reviews of health inequalities, but their policy-making influence is limited. Their reports tend to be focused on concrete knowledge fields, such as gender influence [62], the effects of economic crises [63], or access to healthcare [64]; which are also relevant for the study of health inequalities but whose authorship does not fit the selection criteria, as the main interest of this paper is identifying the health inequalities indicators used by health agencies or similar government-related entities. The reports not produced by this kind of organisation were rejected.

Lastly, *to have a socioeconomic and politic status similar or highly related to Catalonia* criteria was intended to exclude reports whose health indicators were adapted to least developed/developing countries where, for example, access to treatments of diarrhea for infants may still be an issue [65]. Hence, some reports were discarded due to significant differences in the socioeconomic profile of the countries they are studying in comparison to Catalonia or Spain.

These criteria were applied to all the reports and studies found after an extensive search. The addition of country/region names in the search responded to the need of knowing how particular regions of interest were dealing with health inequalities. Interest in regions was mainly based on previous knowledge of concrete public health organizations studying those regions, as well as interest in looking for other organizations in charge of tackling health inequalities in regions similar to Catalonia. Nevertheless, as in any review, it is not possible to ensure that absolutely all the reports suitable for this study were found during the search, nor that they were selected after applying the selection criteria.

### Grouping health indicators into topics

As stated above, the most frequent health indicators were grouped into topics according to the health domain they were measuring (with each indicator related to only one topic). For example, although “*Percentage of 15-year-olds who were overweight in 2009–10, EU Member States by sex*” [25] and “*Obesity rate by body mass index (BMI) (sdg_02_10)*” [23, 66] are different indicators per se, they are both intended to measure the same health issue and, hence, were grouped under the same topic “Obesity and overweight (BMI)” under the indicator name “Obesity and/or overweight (total, by sex, age, or educational level)”.

Most of the selected health indicators were taken from the official statistics of different countries or international organisations, whose development and methodology has been closely consolidated over many years and respond to international standards. In addition, most of these indicators are related to relevant knowledge areas for the study of health inequalities, such as lifestyle habits, deprivation, and mortality*.*

To prioritise the most relevant topics, all the groups with less than five health indicators were deleted. This meant that, unfortunately, interesting topics such as the “Years of potential life lost” [34, 36, 42], “Unmet health needs” [23, 26, 39] and “Passive smokers” [33, 34] were not taken into consideration. Nevertheless, this selection does not imply per se a periodic monitoring of the selected health indicators, as specific topics not present in this list may be studied according to ultimate needs.

### Relevance of the most common topics

All the topics aim to measure and study the relation between determinants of health and health outcomes. Interestingly, three of the top five topics in the list (see Table 2) are related to mortality: life expectancy, infant mortality, and mortality rate. Life expectancy at birth is an indicator of mortality conditions and, by proxy, of health conditions [44]. Hence, life expectancy as well as other mortality-related topics are widely used in the study of health inequalities.

Living and working conditions, such as BMI or smoking, are also key factors in the study of health inequalities as both share a strong socioeconomic gradient [54, 56]. Therefore, as may be expected, they appeared among the top 10 positions in the list of topics (see Table 2). According to our review, the most common way to measure socioeconomic status is to analyse the unemployment rates and material deprivation level of the population.

In Spain, some regions use less common health indicators for studying health inequalities that may be interesting for particular knowledge areas. For example, the Valencian Observatory of Health uses the “Caregiver profile”, which they report to be mostly women, without primary studies, 57-years-old on average, and reporting bad self-perceived health. In addition, they also use “Reasons why contraceptive methods are not used by age and nationality of women” as well as a “Sexual-health information resources (school, parents, friends, etc.)”, which may help to understand possible sexual health inequalities [43]. In the Andalusian School of Public Health, the indicator “Psychosis and mental illnesses due to drugs or alcohol abuse” may be helpful to estimate various negative health outcomes of alcohol and substance abuse that the healthcare system will need to address [41]. Even so, more than a half of topics in Table 2 appear in their reports.

As may be expected, the health indicators used in other reports produced by the Catalan Health System Observatory, such as the “Community Health Indicators”, match the implicit measurement concept behind many of the topics: obesity and overweight, mortality by age (including infant), self-perceived health, and population with primary studies are some of them [67].

### Health indicators

As can be seen in Table 3, health indicators were combined within each topic if stratifiers such as population sex, age, or region were the only difference in their calculation methodologies. Some indicators were also merged if they were formerly different in the way they expressed the same data (i.e., raw number, rate per 1000 or 100,000).

**Table 3:** List of indicators by topic

Topics	Indicators	Reference
Life expectancy	Life expectancy at birth or at certain age (total, by sex, educational level and/or socioeconomic status)	[23–30, 33–36, 38, 41–43]
	Health-adjusted life expectancy	[33, 34]
	Inequality in life expectancy, slope index of inequality (SII) for male and female life expectancy	[35, 39]
	Healthy life years (total, by sex, region and/or socioeconomic status)	[25, 26, 36, 37, 39, 43]
	Slope index of inequality for male and female disability-free life expectancy	[35]
Infant mortality	Infant mortality (total, by sex, by socioeconomic status, deprivation or disposable family income)	[24–30, 33, 34, 36, 38, 40–42]
	Infant mortality of newborns weighting at least 500 g	[34]
	Inequality in infant mortality	[39]
	Highest and lowest infant mortality rates per 1000 live births and measures of inequality between EU Member States, 2000–2010	[25]
Obesity and overweight (BMI)	Obesity and/or overweight (total, by sex, age, or educational level)	[22, 24, 25, 27, 29–33, 36, 38, 42, 43]
	Body mass index	[24]
Mortality rate	All-cause mortality (total, by sex, age, or region)	[25, 27, 29, 34, 37, 38, 41–43]
	Death rates by cause of death	[26, 29, 40, 42]
	% completeness of death registration with cause-of-death information	[30]
Regular smokers /tobacco consumption	Smoking/tobacco consumption (total, by frequency of consumption, age, sex, employment and occupational status and/or by difficulties experienced in paying bills)	[23–25, 27, 29, 31–34, 38, 42]
	Tobacco and alcohol consumption	[43]
	Pregnant women smoking	[24]
Self-perceived health	Self-perceived health (total, by age and/or sex)	[23–26, 31, 32, 34, 37, 38, 42, 43]
	Total health gain as assessed by patients for elective procedures: physical health related procedures/psychological therapies	[36]
Unemployment	Unemployment (total, by duration/long-term)	[24, 25, 27, 28, 30, 38, 42]
	Employment of people with long-term conditions/mental illness/disabilities	[36, 41]
	Eligibility for employment insurance (aged 15–69)	[34]
	Age dependency ratio (% working-age population)	[30]
	Population living in jobless households	[39]
	Employment gap	[39]
Mental well-being	Risk of psychological suffering	[42]
	Health-related quality of life for people with mental illness (eventual or recovery)	[36]
	Psychological well-being or discomfort (by age, by using GHQ-12)	[24, 37, 43]
	Mental disorders or illness (ICD9MC: 290–319 / including addictions)	[39, 41]
	Morbidity: neurotic, personality, and other non-psychotic mental disorders (except drugs or alcohol) (ICD9MC: 300–302, 306–319)	[41]
	Depression (mental health)	[38]
	Mental illness hospitalisation rate (total, by age)	[33, 34]
	Coverage of services for severe mental health disorders	[29]
Cardiovascular disease/ hypertension	Mortality due to cardiovascular causes, including heart diseases (total, by sex and/or age)	[27, 34, 36, 37, 43]
	Arterial hypertension	[32, 38, 41]
	Blood pressure	[24, 29]
	Proportion of stroke patients reporting an improvement in activity	[36]
	Cardiovascular and heart diseases (including heart attacks, angina pectoris, and heart failure)	[38]
	First ever hospital admission for heart attack (aged under 75 years)	[37]
	30-day in-hospital case-fatality AMI and stroke	[24]
Socioeconomic status (SES)/ material deprivation	Working poor	[33, 34, 39]
	Disposable family income/family SES	[39, 42]
	Income tax base	[38]
	People in households in receipt of means-tested benefits	[35]
	Slope index of inequality for people in households in receipt of means-tested benefits	[35]
	% at risk of poverty – with less than 60% of the median income/% at persistent risk of poverty (by intensity of poverty)	[39]
	% who own a house and car	[39]
	% areas with > 20% population poor	[39]
	Age-standardised percentage of people aged 25 and over by severity of material deprivation	[25]
	SII Income/SII Deprivation (by perceived health)/inequality relative index/Gini coefficient (income distribution)/salary gap/income inequality (S80/S20) within and across local areas	[25, 28, 39]
	Population below poverty line and income inequality	[24]
Diabetes / Insulin resistance	Age-standardised prevalence rate of anti-diabetic drug recipients	[38]
	Diabetes (excluding gestational) (by region, age)	[25, 30, 32–34, 41]
	Raised blood glucose/diabetes among adults	[29]
	Diabetes control	[24]
Physical activity	Physical activity, active or moderately active (total or during free time, by sex and/or age)	[24, 31, 34, 38]
	Sedentarism/insufficient physical activity	[29, 32, 42]
	% children by the number of hours of physical activity during a week	[38]
	Little social or recreational activity	[39]
Cancer	Cancer mortality (total, by sex, age)	[27, 36, 37]
	Cancer incidence (total, by age)	[25, 34, 37]
	Lung cancer incidence or mortality	[33, 34, 43]
	Survival rates cancer (1–5 years from all cancers/diagnosed at stages 1 and 2)	[24, 36]
	Colorectal cancer screening, past 5 years (aged 50–74)	[34]
HIV	HIV incidence / prevalence (total, by sex, age)	[24, 27, 29, 30, 34, 40, 41]
	AIDS-related mortality rate	[29, 40]
	People living with HIV who know their status	[29]
	Antiretroviral therapy coverage	[29]
	Prevention of mother-to-child transmission	[29]
	HIV test results for TB patients (positive results)	[29]
	Death rate due to TB, HIV, and hepatitis by sex	[23]
Long-term limitations/ chronic illnesses	Health-related quality of life for people with long-term conditions	[36]
	Long-term/chronic conditions, limitations, or illness (total, by age, sex)	[24, 25, 31, 34, 37]
	Self-reported chronic morbidity or limitations in daily activities	[24, 26]
	Physical and sensory functional limitations	[24]
	Death rate due to chronic diseases by sex	[23]
Tuberculosis	Incidence/prevalence of TB (by country of origin, nationality, age, and sex)	[29, 30, 33, 34, 40, 42, 43]
	Evolution of anti-TB vaccination (BCG)	[40]
	TB notification rate	[29]
	Coverage for TB treatment/drug susceptibility testing (for active, latent infection or drug-resistant)	[29]
	Death rate due to TB, HIV, and hepatitis by sex	[23]
Hazardous alcohol consumption	Hazardous alcohol consumption/heavy drinking (total, by age)	[24, 33, 34, 38]
	Alcohol-related deaths (aged 45–74 years)	[37]
	High-risk alcohol consumption	[32]
	Harmful use of alcohol, defined according to the national context as alcohol per capita consumption (aged 15 years and older) within a calendar year in litres of pure alcohol	[29]
Low birthweight	Low birthweight	[27, 29, 34, 37, 38, 42]
	Preterm delivery	[29, 38]
	Hospital discharges due to delayed intrauterine growth, foetal malnutrition, shortened pregnancy and low birth weight, caesarean sections, and low birth weight infants (by province)	[41]
	Small for gestational age	[34]
Perinatal, neonatal, and stillbirths/foetal mortality	Perinatal mortality	[24, 27, 43]
	Neonatal mortality rate	[29, 30, 36]
	Under-5 mortality rate	[29, 30]
	Stillbirth mortality	[29, 36]
	Foetal mortality by nationality	[43]
	Stillbirths, perinatal mortality, and infant mortality	[38]
General practitioner (GP) utilisation	Health professionals (including doctor/GP/specialist) consultations (by time period)	[31, 32, 34]
	Care utilisation (including GP)	[24, 26]
	Experience of GP services/Out of Hours service	[36]
	% without a GP	[39]
Suicide/self-harm	Suicide mortality	[25, 29, 33, 34, 38]
	Suicide and mortality from injury of undetermined intent among people with recent contact from NHS	[36]
	Suicides and self-injuries	[43]
Healthcare resources	Hospital beds	[24, 27, 30]
	Health facilities	[40]
	% areas understaffed in health & education	[39]
	Physicians employed (total or rate, by region)	[24, 27, 38, 40]
	Specialist surgical workforce (per 100,000 population)	[30]
	Nurses employed including/excluding midwives (total and rate)	[24, 27, 40]
Alcohol consumption	Alcohol consumption (total, by sex)	[24, 31, 38]
	Alcohol first hospital admissions (aged under 75 years)	[37]
	Alcohol risk	[32]
	Patterns of alcohol consumption	[25]
Road traffic accidents (injuries and deaths)	Mortality caused by road traffic injury (per 100,000 people)	[29, 30]
	Healthy life years lost by traffic accidents and falls	[38]
	Road injuries and deaths (register-based and self-reported)	[23, 24, 27]
Food consumption (vegetables, fruit, salt)	Fruit/vegetable consumption (total, by times a day, by age)	[24, 34, 38]
	Salt intake	[29, 32]
	Low access to healthy food	[39]
Primary studies/ illiteracy	Population by education (including early school leavers)	[24, 28, 39, 42]
	Young people who are not in education, employment or training	[35]
	Days away from study or work	[32]
	Results in math and literacy or years of education	[39]
	% illiterate or does not know the language well	[39]
Child well-being	Hospital discharges in girls and boys by age	[41]
	Children well-being/achieving a good level of development at age 5	[35, 39]
	Early childhood development	[33]
	Incidence of one of the 17 most common disorders in children, by sex and age	[31]
Respiratory disease	Mortality rate from respiratory disease (including COPD, total, by age)	[36, 38]
	COPD and associated diseases (ICD9MC: 490-496)	[24, 41]
	Bronchitis and acute bronchiolitis including emphysema (ICD9MC: 466)	[32, 41]
	Care-seeking for symptoms of pneumonia	[29]
Work-related health risks	Health-related quality of life for carers	[36]
	Occupational diseases	[43]
	Work accidents	[39]
	Health worker density and distribution	[29]
	Work-related health risks	[24]
	People killed in accidents at work	[23]
Dental care/ oral health	Dental consultations	[26, 32, 34]
	Tooth extractions in secondary care for children under 10	[36]
	Dental care (regular brushing of teeth, regular visits to the dentist, proper diet, and the use of protective agents)	[38]
	Dental disease (caries and periodontal disease)]	[36, 38]
	Dental pain or discomfort, past month (aged 18+)	[34]
	Inability to chew	[33, 34]
	Decay-missing-filled teeth index (aged 6–17)	[34]
Policy and legislation	A measure of the effectiveness of post-diagnostic care in sustaining independence and improving quality of life	[36]
	New cases of International Health Regulations (IHR)-notifiable diseases and other notifiable diseases	[29]
	Total net official development assistance to medical research and basic health sectors prepared	[29]
	International health regulations capacity and health emergency preparedness	[29]
	Integrated programmes in settings, including workplace, schools, hospital	[24]
	Expenditure on public health administrations	[40]
	Legislation, plans and funds to fight discrimination and structural health inequalities	[28, 39]
	Prevention of HIV in key populations	[29]
	Policies and practices on healthy lifestyles including nutrition	[24]
Perceived mental health	Psychological distress (total or by place, by age)	[24, 30, 34]
	Excess under 75 mortality rates in adults with common mental illness	[36]
	Perceived mental health (fair or poor)	[33, 34]
Pregnancy care/ breastfeeding	Breastfeeding (total, having ever breastfed, initiation, exclusive, by age)	[24, 32, 34]
	Recommended duration of breastfeeding	[32]
	Hospital discharge for giving birth (ICD9MC: 650)	[41]
	% pregnant women receiving prenatal care	[39]
Hip fractures and surgical procedures	Proportion of patients with hip fractures recovering to their previous levels of mobility/walking ability at 30 days	[36]
	Fractures including hip, vertebral and forearm fractures (ICD9MC: 800–829) (by province, sex, age)	[41, 43]
	Number of surgical procedures including PTCA, hip, cataract (per 100,000 population)	[24, 30]

The health indicators within each topic often cover a different aspect relevant to health inequalities. For example, in the topic “Tuberculosis” [23, 29, 33, 34, 40, 42, 43] indicators about incidence, prevalence, or mortality can be observed. In addition, health indicators about treatment coverage or vaccination are also included in this topic. Overall, in most topics, indicators try to measure every relevant (and measurable) aspect of the topic.

Common stratifiers are sex, age, and studied region, something that is coherent with the determinants of health perspective and the focus on inequalities. However, the stratifiers found are highly heterogeneous and may also include socioeconomic status, educational level, or nationality/country of origin, among many others.

### Research fitted to monitor health inequalities

This comprehensive review was carried out to help accomplish the first step in the process for tackling and monitoring health inequalities: selecting high-impact issues and health indicators [19, 20]. The next steps in the analysis will be to carefully take into account stratifiers such as area of residence, gender, age, and nationality. Lastly, after the identification of key health inequalities, decision-making stakeholders will need to play a role during the last steps: determining priorities of action and implementing changes. The time variable will play a key role in the monitoring, as it will indicate the possible health consequences of policy-making decisions [19, 20].

## Conclusions

Reviewing the most common health indicators and topics used in the study of health inequalities may help research teams in different ways. First, having an overview of what is being done by their neighbouring countries or regions may highlight issues that should not be missed when selecting relevant health topics to study. Second, even if some topics might not ultimately be chosen as a priority of action, having a complete list of key issues will provide an overview of what is relevant in the study of health inequalities, as well as some interesting insights. Lastly, knowing what other research institutions are working on will promote potential collaborations between organizations, creating synergies and bonds that may lead to better understanding and monitoring of health inequalities.

At a regional level, these results are highly valuable for the first stages of health inequalities monitoring cycles in Catalonia. This study provided the basis for choosing health topics to study as well as helped gain insights about which indicators should be used. In addition, regions with similar socioeconomic status and goals in tackling health inequalities may benefit from this research. Similarly, at a national and international level these results may help organizations shift the focus towards undermined health inequalities topics or explore new areas of knowledge (yet unstudied or with a different perspective).

## Data Availability

No analysis of quantitative data was performed. Hence, data availability declaration is not applicable.

## Abbreviations

AIDS: Acquired immune deficiency syndrome
AMI: Acute myocardial infarction
BCG: Bacillus Calmette-Guérin
BMI: Body mass index
COPD: Chronic obstructive pulmonary disease
CRDSS-E: Comisión para Reducir las Desigualdades Sociales en Salud en España
CVD: Cardiovascular disease
ECHI: European Core Health Indicators
GHQ-12: 12-Item General Health Questionnaire
GP: General practitioner
HI: Health indicators
HIV: Human Immunodeficiency Virus
HLY: Healthy Life Years
ICD9MC: International Classification of Diseases, Clinical Modification
IHR: International Health Regulations
NHS: National Health Service
PDF: Portable document format
PHAC: Public Health Agency of Canada
PINSAP: Interdepartmental and Intersectorial Public Health Plan
PTCA: Percutaneous transluminal coronary angioplasty
SDG: Sustainable Development Goals
SES: Socioeconomic status
SII: Slope index of inequality
TB: Tuberculosis
URL: Uniform resource locator
UN: United Nations
WHO: World Health Organization

## References

[1] World health organization (WHO) (2011). Meeting report of the world conference on social determinants of health. Rio de Janeiro, 19-21.

[2] Comisión para Reducir las Desigualdades Sociales en Salud en España. Propuesta de políticas e intervenciones para reducir las desigualdades sociales en salud en España. Gac Sanit. 2012:182–9. 10.1016/j.gaceta.2011.07.024 22112713

[3] Comisión para Reducir las Desigualdades Sociales en Salud en España. Análisis de situación para la elaboración de una propuesta de políticas e intervenciones para reducir las desigualdades sociales en salud en España. Madrid: Ministerio de Sanidad y Política Social; 2009. http://www.mscbs.gob.es/profesionales/saludPublica/prevPromocion/promocion/desigualdadSalud/docs/Analisis_reducir_desigualdes.pdf. Accessed 24 July 2019.

[4] Comisión para Reducir las Desigualdades Sociales en Salud en España. Avanzando hacia la equidad: propuesta de políticas e intervenciones para reducir las desigualdades sociales en salud en España. Madrid: Ministerio de Sanidad y Política Social; 2010. http://www.mscbs.gob.es/profesionales/saludPublica/prevPromocion/promocion/desigualdadSalud/docs/Propuesta_Politicas_Reducir_Desigualdades.pdf. Accessed 24 July 2019.

[5] World Health Organization (WHO) (2011). Rio political declaration on social determinants of health Rio de Janeiro, Brazil.

[6] Working Group for Monitoring Action on the Social Determinants of Health (2018). Towards a global monitoring system for implementing the Rio Political Declaration on Social Determinants of Health: developing a core set of indicators for government action on the social determinants of health to improve health equity. Int J Equity Health.

[7] Government of Catalonia (2016). Health plan for Catalonia 2016–2020.

[8] Comissió Interdepartamental de Salut (2017). Pla interdepartamental i intersectorial de salut pública.

[9] Cabezas-Peña C (2018). Catalonia, Spain. Regions for Health Network (RHN).

[10] Observatori del Sistema Salut de Catalunya (2014). Efectes de la crisi econòmica en la salut de la població de Catalunya.

[11] Observatori del Sistema Salut de Catalunya (2015). Efectes de la crisi econòmica en la salut de la població de Catalunya. Anàlisi territorial.

[12] Observatori del Sistema Salut de Catalunya (2017). Desigualtats socioeconòmiques en la salut i la utilització de serveis sanitaris públics en la població de Catalunya.

[13] Observatori del Sistema Salut de Catalunya (2014). Central de Resultats: Efectes de la crisi econòmica en la població infantil de Catalunya.

[14] Observatori del Sistema Salut de Catalunya (2008). Central de Resultats: Evolució de la utilització de serveis i el consum de fàrmacs 2008–2015.

[15] García-Altés A, Ruiz-Muñoz D, Colls C, Mias M, Martín BN (2018). Socioeconomic inequalities in health and the use of healthcare services in Catalonia: analysis of the individual data of 7.5 million residents. J Epidemiol Community Health.

[16] Ruiz-Muñoz D, Colls C, Mias M, Martín N, García-Altés A (2017). Desigualtats socioeconòmiques en la salut i la utilització dels serveis sanitaris públics en la població de Catalunya. Ann Med.

[17] Genoveva B, Ruiz-Muñoz D, García-Altés A (2016). Efectes de la crisi econòmica en la salut de la població de Catalunya: anàlisi territorial. Ann Med.

[18] Health for Everyone?. OECD Health Policy Studies. Paris: OECD; 2019. https://www.oecd-ilibrary.org/social-issues-migration-health/health-for-everyone_3c8385d0-en. Accessed 9 Jan 2021.

[19] World Health Organization (WHO) (2013). Handbook on health inequality monitoring with a special focus on low- and middle-income countries.

[20] UK Department of Health. Health equity audit: a self-assessment tool. London: Department of Health, 2004: 25. https://webarchive.nationalarchives.gov.uk/20120105214155/http://www.dh.gov.uk/en/Publicationsandstatistics/Publications/PublicationsPolicyAndGuidance/DH_4070715. Accessed 13 Sept 2019.

[21] WHO Regional Office for Europe. Health 2020: A European policy framework and strategy for the 21st century. Geneva: WHO; 2013:190. http:// www.euro.who.int/__data/assets/pdf_file/0011/199532/Health2020-Long.pdf. Accessed 24 July 2019.

[22] Pencheon D (2017). The good indicators guide: understanding how to use and choose indicators.

[23] Eurostat. SDG 3. Good health and well-being. Luxembourg: European Commission. https://ec.europa.eu/eurostat/web/sdi/good-health-and-well-being. Accessed 8 Apr 2020.

[24] ECHI-European Core Health Indicators. Luxembourg: European Commission. https://ec.europa.eu/health/indicators/echi/list_en#id3. Accessed 8 Apr 2020.

[25] Marmot M (2013). Health inequalities in the EU - final report of a consortium.

[26] Social Protection Committee (2015). Indicators Sub-group. Portfolio of EU social indicators for the monitoring of progress towards the EU objectives for social protection and social Inclusion.

[27] I2sare project. Galicia, Spain profile. Regional Health Profiles in the European Union, 2010. www.sergas.es/Saude-publica/-I2SARE-Galicia. Accessed 8 Apr 2020.

[28] World Health Organization (WHO). Data Management Tool. Copenhagen: WHO Regional Office for Europe. http://dmt.euro.who.int/classifications/tree/B#B02. Accessed 8 Apr 2020.

[29] World Health Organization (WHO) (2018). 100 core health indicators (plus health-related SDGs).

[30] The World Bank. Health. Data. Washington DC: The World Bank. https://data.worldbank.org/topic/health. Accessed 8 Apr 2020.

[31] Observatori Social d'Andorra. Sant Julià de Lòria (Andorra): Centre d’Estudis Andorrans. Govern d'Andorra https://observatorisocial.ad/index.php. Accessed 8 Apr 2020.

[32] Turrell G, Stanley L, de Looper M. Oldenburg B. Health inequalities in Australia: morbidity, health behaviours, risk factors and health services use (AIHW). Canberra; 2006. www.aihw.gov.au/getmedia/0cbc6c45-b97a-44f7-ad1f-2517a1f0378c/hiamhbrfhsu.pdf. Accessed 8 Apr 2020

[33] Public Health Agency of Canada (2018). Key Health Inequalities in Canada: A National Portrait.

[34] Canadian Institute of Health Information. Health Inequalities Data Tool. Ottawa: Government of Canada https://health-infobase.canada.ca/health-inequalities/data-tool/index. Accessed 8 Apr 2020.

[35] Institute of Health Equity (2017). Marmot indicators release 2017.

[36] NHS England Analytical Services & the Equality and Health Inequalities Unit (2016). England Analysis: NHS Outcome Framework Health Inequalities Indicators.

[37] Scottish Government (2018). Long-term monitoring of health inequalities.

[38] Buzeti T, Djomba JK, Blenkuš MG, Ivanuša M, Klanšček HJ, Kelšin N (2011). Health inequalities in Slovenia.

[39] Ministry of Health and Social Policy of Spain (2010). Moving forward equity in health: monitoring social determinants of health and the reduction of health inequalities.

[40] Instituto Nacional de Estatística-Statistics Portugal (2012). Indicadores Sociais 2011.

[41] García-Calvente MM, del Río LM, Marcos-Marcos J (2015). Guía de indicadores para medir las desigualdades de género en salud y sus determinantes.

[42] Malmusi D (2017). Desigualtats en salut, respostes a nivell local: Polítiques per reduir les desigualtats en salut a la ciutat de Barcelona.

[43] Observatorio Valenciano de la Salud (2018). Desigualdades en Salud en la Comunidad Valenciana.

[44] United Natios (UN). Life expectancy at birth. New York: UN. http://www.un.org/esa/sustdev/natlinfo/indicators/methodology_sheets/health/life_expectancy.pdf. Accessed 19 Mar 2020.

[45] Shryock HS, Siegel JS, Stockwell EG (1976). The methods and materials of demography.

[46] Global Health Observatory (GHO) data (2018). Infant mortality.

[47] Porta M (2016). A dictionary of epidemiology, sixth edition.

[48] World Health Organization (WHO). Cancer. Geneva: WHO. http://www.who.int/health-topics/cancer#tab=tab_1. Accessed 7 Apr 2020.

[49] World Health Organization (WHO). Diabetes. Geneva: WHO. http://www.who.int/health-topics/diabetes#tab=tab_1. Accessed 7 Apr 2020.

[50] World Health Organization (WHO). HIV/AIDS. Geneva: WHO. http://www.who.int/health-topics/hiv-aids#tab=tab_1. Accessed 7 Apr 2020.

[51] World Health Organization (WHO). Tuberculosis. Geneva: WHO. http://www.who.int/health-topics/tuberculosis#tab=tab_1. Accessed 7 Apr 2020.

[52] Bambra C, Gibson M, Sowden A, Wright K, Whitehead M, Petticrew M (2010). Tackling the wider social determinants of health and health inequalities: evidence from systematic reviews. J Epidemiol Community Health.

[53] Organisation for Economic Co-operation and Development (OECD) (2003). OECD Glossary of Statistical Terms-Unemployed–ILO Definition.

[54] Pigeyre M, Rousseaux J, Trouiller P, Dumont J, Goumidi L, Bonte D (2016). How obesity relates to socio-economic status: identification of eating behavior mediators. Int J Obes.

[55] World Health Organization (WHO). Obesity and overweight. Geneva: WHO. http://www.who.int/en/news-room/fact-sheets/detail/obesity-and-overweight. Accessed 7 Apr 2020

[56] WHO (2014). Tobacco and inequities Guidance for addressing inequities in tobacco-related harm Written by: Belinda Loring.

[57] World Health Organization (WHO). Harmful use of alcohol. Geneva: WHO. http://www.who.int/health-topics/alcohol#tab=tab_1. Accessed 7 Apr 2020.

[58] World Health Organization (WHO). Physical activity. Geneva: WHO. http://www.who.int/news-room/fact-sheets/detail/physical-activity. Accessed 7 Apr 2020.

[59] American Psychological Association (APA). Socioeconomic Status. Washinton DC. http://www.apa.org/topics/socioeconomic-status/. Accessed 6 Apr 2020.

[60] Eurostat. Glossary: Material deprivation - Statistics Explained. Luxembourg: European Commission. https://ec.europa.eu/eurostat/statistics-explained/index.php/Glossary:Material_deprivation. Accessed 6 Apr 2020.

[61] Solar O, Irwin A (2010). A conceptual framework for action on the social determinants of health. Social Determinants of Health Discussion Paper 2.

[62] Salcedo N, Saez M, Bragulat B, Saurina C (2012). Does the effect of gender modify the relationship between deprivation and mortality?. BMC Public Health.

[63] Regidor E, Barrio G, Bravo MJ, de la Fuente L (2014). Has health in Spain been declining since the economic crisis?. J Epidemiol Community Health.

[64] Consulting Services Limited ICF (2018). Towards a fairer and more effective measurement of access to healthcare across the EU final.

[65] Ebinger JO, Hamso B, Gerner F, Lim A, Plecas A (2008). Europe and Central Asia region.

[66] Eurostat (2018). Eurostat - Tables, Graphs and Maps Interface (TGM) table.

[67] Catalan Healthcare System Observatory (2017). Indicadors de salut comunitària.

